# CC3/TIP30 affects DNA damage repair

**DOI:** 10.1186/1471-2121-11-23

**Published:** 2010-04-07

**Authors:** Sylvia Fong, Frank King, Emma Shtivelman

**Affiliations:** 1 BioNovo Inc, 5858 Horton Street, Emeryville 94608, CA, USA

## Abstract

**Background:**

The pro-apoptotic protein CC3/TIP30 has an unusual cellular function as an inhibitor of nucleocytoplasmic transport. This function is likely to be activated under conditions of stress. A number of studies support the notion that CC3 acts as a tumor and metastasis suppressor in various types of cancer. The yeast homolog of CC3 is likely to be involved in responses to DNA damage. Here we examined the potential role of CC3 in regulation of cellular responses to genotoxic stress.

**Results:**

We found that forced expression of CC3 in CC3-negative cells strongly delays the repair of UV-induced DNA damage. Exogenously introduced CC3 negatively affects expression levels of DDB2/XPE and p21CIP1, and inhibits induction of c-FOS after UV exposure. In addition, exogenous CC3 prevents the nuclear accumulation of P21CIP in response to UV. These changes in the levels/localization of relevant proteins resulting from the enforced expression of CC3 are likely to contribute to the observed delay in DNA damage repair. Silencing of CC3 in CC3-positive cells has a modest delaying effect on repair of the UV induced damage, but has a much more significant negative affect on the translesion DNA synthesis after UV exposure. This could be related to the higher expression levels and increased nuclear localization of p21CIP1 in cells where expression of CC3 is silenced. Expression of CC3 also inhibits repair of oxidative DNA damage and leads to a decrease in levels of nucleoredoxin, that could contribute to the reduced viability of CC3 expressing cells after oxidative insult.

**Conclusions:**

Manipulation of the cellular levels of CC3 alters expression levels and/or subcellular localization of proteins that exhibit nucleocytoplasmic shuttling. This results in altered responses to genotoxic stress and adversely affects DNA damage repair by affecting the recruitment of adequate amounts of required proteins to proper cellular compartments. Excess of cellular CC3 has a significant negative effect on DNA repair after UV and oxidant exposure, while silencing of endogenous CC3 slightly delays repair of UV-induced damage.

## Background

The human gene CC3/TIP30 was originally identified as a metastasis-suppressor of variant small cell lung carcinoma (vSCLC) [1]. CC3 is a phylogenetically conserved protein whose expression is absent or much reduced in a variety of aggressive or metastatic tumors such as vSCLC [1], neuroblastoma and glioblastoma [2,3], metastatic breast cancer [4], gastric cancer [5], hepatocellular carcinoma [6,7], colorectal carcinomas [8] and lung cancers with poor prognosis [9]. Forced expression of CC3 in vSCLC [1], mouse melanoma, breast carcinoma [10], hepatocellular carcinoma [6] and gastric carcinoma cell lines [5] inhibits metastatic behavior *in vitro *and/or metastasis *in vivo*. The deletion of CC3 in germline results in spontaneous tumorigenesis in mice [11], and CC3-null mammary epithelial cells undergo immortalization in vitro [12] indicating that CC3 could be not only a metastasis suppressor, but also a tumor suppressor [13]. One study on the role of TIP30 in metastasis reported that TIP30 expression actually enhances growth and metastatic behavior of prostate carcinoma cells in vitro [14], but majority of published results support the hypothesis that CC3/TIP30 suppresses tumor development and metastasis.

High levels of acutely overexpressed exogenous CC3 induce apoptosis [1,3] while stable expression of exogenous CC3 results in sensitization of cells to apoptosis after treatment with a variety of factors such as serum withdrawal, cytotoxic drugs, γ-irradiation, and microtubule poisons [3]. Expression of CC3 in CC3-negative tumor cells has an inhibitory effect on the ability of these cells to produce angiogenic factors *in vitro *[2], consistent with the conclusion that down regulation of CC3 contributes to the development of aggressive metastatic phenotypes.

The precise cellular function of CC3 remains obscure. A significant sequence homology was reported between CC3 and short-chain dehydrogenases-reductases or SDRs [15,16]. CC3 sequence contains a domain, well conserved between CC3 and SDR enzymes, that was predicted to serve as a NADP(H) binding site [15,16], and the structural analysis of CC3 protein confirmed this prediction [17]. A clue to the potential cellular function of CC3 came from the findings that CC3 plays an unanticipated inhibitory role in the regulation of nuclear transport [18]. CC3 binds directly to the karyopherins of the importin β family in a RanGTP-insensitive manner and associates with nucleoporins *in vivo*. CC3 inhibits nuclear import of proteins with either the classic nuclear localization signal (NLS) recognized by importin α:β_1_, or the M9 signal recognized by transportin (importin β2). Cells modified to express higher levels of CC3 are predisposed to apoptosis and have a slower rate of nuclear import [18]. CC3 protein with mutated NADP(H) binding site lacks pro-apoptotic activity, is displaced from transportin by RanGTP, and fails to inhibit nuclear import *in vitro *and *in vivo*. Our results suggest that the ability of CC3 to form a RanGTP resistant complex with importins and the NPC is central to its ability to inhibit nuclear import and induce apoptosis [18].

An independent confirmation for the function of CC3 as a negative regulator of nuclear transport came from other studies. CC3 was shown to be aberrantly expressed in the oligodendrocyte precursors found in the lesions associated with multiple sclerosis. These high levels of CC3 cause the arrest of the nuclear import of the intracellular domain of Notch (NICD). In these cells NCID, rather that accumulating in the nuclei, is sequestered in cytoplasm in a complex with importin and CC3/TIP30 [19]. The observed lack of the nuclear translocation of NICD leads to the failure of remyelinaiton that could play a causative role in pathogenesis of multiple sclerosis [19].

The function of CC3 in nuclear transport is likely to be evolutionarily conserved. A large scale two-hybrid analysis of the yeast proteome uncovered that the S. cerevisiae homologue of CC3, YER004w, interacts with exportin CRM1 and with NTF2 [20], the import factor for RanGDP. Global analysis of yeast transcriptome changes after exposure to DNA damaging treatments strongly indicates that CC3 might be also involved in the DNA damage responses. The yeast homologue of CC3, YER004w belongs to a small group of nine genes that are known as "DNA damage signature set" [21]. Transcription of this group of genes, including YER004w, is increased significantly and specifically after several different types of DNA damage in a MEC1 (ATR homologue)-dependent manner [21]. Additionally, six of the genes in the DNA damage signature set, among them YER004w, are expressed at higher levels in the dna2-1 replication mutant that undergoes premature aging most likely due to the spontaneously accumulating DNA damage [22]. These data, and the possibly conserved role of YER004w in nuclear transport in yeast, indicate that the yeast homologue of CC3 could be involved in control of nuclear transport in response to DNA damage.

We have initiated this work to examine the potential role of human CC3 in regulation of DNA damage responses. We report here that enforced expression of exogenous CC3 significantly impairs the repair of DNA after exposure to UV and oxidative agent as well as negatively influences cell survival, while silencing of endogenous CC3 in human cells has a mild delaying effect on repair of UV induced damage. Our results are consistent with findings that demonstrate a role for CC3 in decreasing cell survival in response to a variety of death signals including DNA damage.

## Results

### Excess of CC3 impairs the repair of DNA damage

For the initial assessment of the potential effects of CC3 on repair of UV induced DNA damage we have employed the host cell reactivation assay (HCR). HCR is a transfection-based assay in which cells repair transfected UV damaged reporter plasmid. Through measurement of the activity of a reporter enzyme, the relative amount of damaged plasmid that a cell can "reactivate" or repair, and express, can be quantified. We have used a firefly luciferase reporter plasmid damaged by UV and a Renilla luciferase plasmid as an internal control of the transfection efficiency. These luciferase plasmids were co-transfected into recipient HeLa cells along with the effector plasmids (empty vector or CC3-expressing), and dual luciferase activity assays were performed 24 hours after transfection.

**Figure 1:**
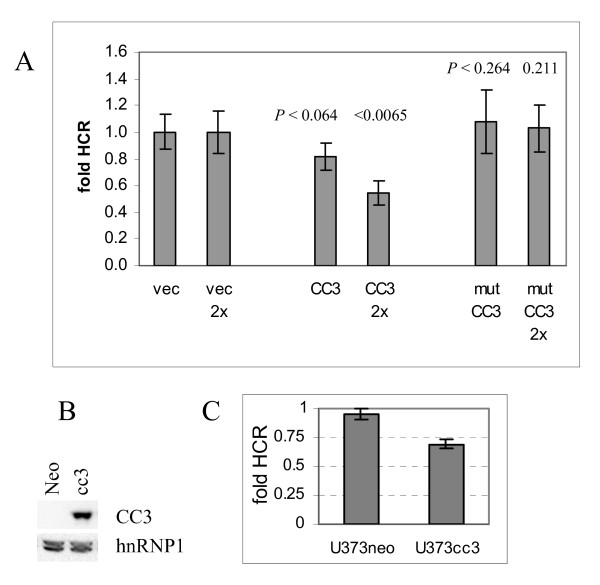
**Host cell reactivation of the UV-damaged plasmid in presence of exogenously expressed CC3**. (A). HeLa cells were transfected as described in Methods with reporter plasmids and either 1× or 2× amount of effector plasmids indicated. Fold HCR in presence of control, wild type CC3 and mutant CC3 plasmids is shown. P values were determined by t-test, n = 3. Shown are the statistical significances of differences seen between 1× amount of CC3 versus 1× amount vector (0.064), 1× mutant CC3 versus vector (0.264) and for 2× amounts of CC3 versus vector (0.0065) and mutant/vector (0.211). (B). Expression of CC3 protein in U373neo and stably transfected U373cc3 cells determined by Western blot analysis of cell extracts using antibody to CC3 [18]. Antibody to hnRNPI was used for loading control. (C). U373 cell clones, control (U373neo) and CC3 expressing (U373cc3) were transfected with luciferase reporter plasmids and analyzed for HCR. All results are average of three transfections performed in duplicates.

CC3 expression vectors for wild type CC3 and the mutant version with a mutation in the NADPH binding site, as well as the empty vector, were used as effector plasmids. The mutant version of CC3 (G28A, G31A) does not bind to importins in a Ran-independent manner, and does not inhibit nuclear transport [18]. Results in Figure 1A show that expression of wild type but not mutant CC3 negatively affects repair of DNA damage. Transfection of twice higher amount of wild type CC3 vector (2×) had a stronger inhibitory effect of the repair of DNA damage (Figure 1A) indicating a dose response relationship between the amounts of CC3 and inhibition of DNA repair.

To confirm the inhibitory role of CC3 in UV induced DNA damage repair, HCR was conducted in CC3-negative glioblastoma cell line U373 stably transfected with exogenous CC3 ([2] and Figure 1B). Out of several CC3-negative transformed cell lines that were stably transfected with CC3 [2], we have chosen the U373 cells because, unlike in other lines, expression of exogenous CC3 had no noticeable effect on the proliferation rate of U373, and did not induce an appreciable increase in their susceptibility to apoptotic stimuli such as UV irradiation or DNA damaging drugs (data not shown; Additional File 1). This allowed monitoring the repair of DNA damage without interference from the different growth rates or from differential induction of cell death by UV. A control clone transfected with empty vector (U373neo) and a clone expressing moderate levels of CC3 (U373cc3) were used in HCR assay with luciferase reporters. Figure 1C shows that in U373neo cells the repair is more efficient that in U373cc3, thus confirming the inhibitory effect of CC3 on DNA damage repair in an additional cellular context.

### Stably expressed exogenous CC3 delays repair of both CPDs and 6-4PPs

**Figure 2:**
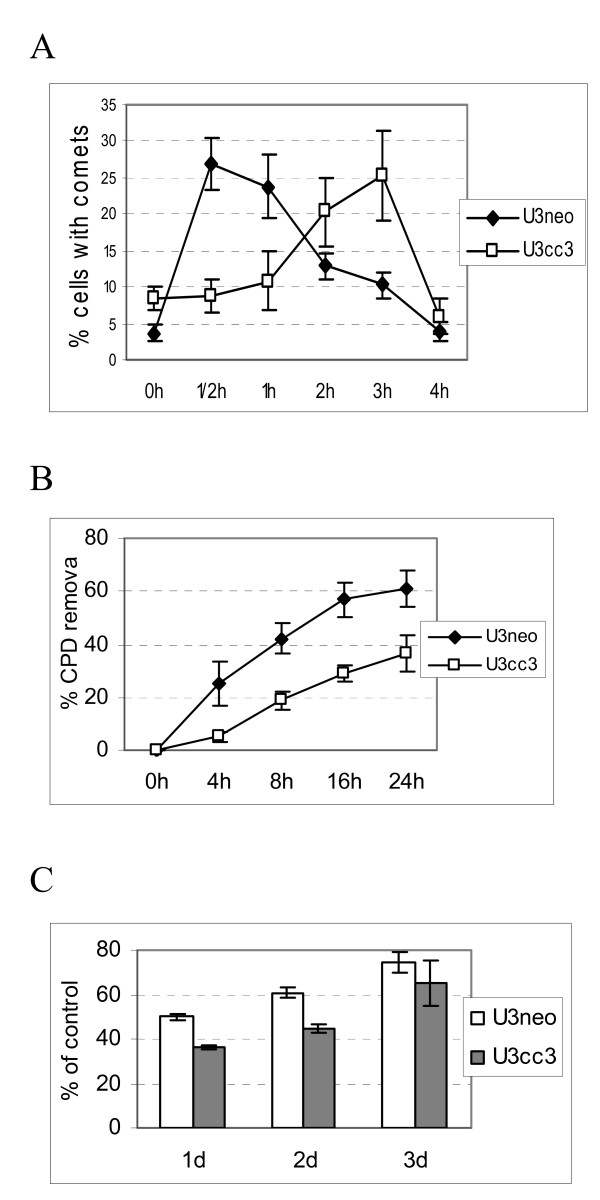
**Delay in repair of UV-induced DNA damage induced by exogenously expressed CC3**. (A). U373 clonal populations, control (U3neo) and stably expressing CC3 (U3cc3), were irradiated with 15 J/m^2 ^UVC and harvested at different times after exposure. After lysis and denaturation, cells were subjected to Comet assay analysis according to the manufacturer's (Trevigen) protocol. (B). Cells were exposed to UV as in A, and the cellular DNA was isolated at the times indicated. The repair of CPD lesions was quantified using ELISA with an anti-CPD antibody. All results are average of three experiments. (C). Proliferation of U373 clones subjected to 15 J/m^2 ^UVC in a short-term assay. Cells were analyzed as described in Materials and methods; results are expressed as percent of untreated cultures; experiments were performed three times with cells plated in triplicate or quadruplicate for each experiment.

Irradiation of cells with UVC light induces two major types of mutagenic DNA photoproducts: cyclobutane pyrimidine dimers (CPD) and pyrimidine (6-4) pyrimidone photoproducts (6-4PP). The latter are repaired relatively quickly, while repair of CPDs can continue for periods well over 24 hours. We have used the comet assay to examine repair of 6-4PP and other fast-repaired lesions such as oxidized nucleotides and apurinic/apyrimidininc bases, while CPDs were detected with a specific antibody by ELISA. Figure 2A shows that repair of 6-4PP is significantly delayed in U373cc3 cells. In control cells the maximum number of breaks, resulting from recognition and excision of damaged bases, was observed at 30 minutes after exposure, whereas in U373cc3 cells the maximum number of comets was observed at 3 hours after exposure. This suggests that the process of damage recognition and/or excision is delayed in U373cc3 cells, while the fill-in synthesis and ligation proceed normally, because by 4 hours after exposure both clones have mostly repaired the fast-repaired UV-induced lesions.

Repair of CPD was monitored over a period of 24 hours. Figure 2B shows that removal of CPD is also significantly delayed in U373cc3 cells, and after 24 hours 60% of CPDs remain unrepaired in DNA from U373cc3 cells versus less than 40% in control cells.

We have examined if delays in DNA damage repair observed in U373cc3 cells have an effect on cell viability or proliferation. Cell death assays (binding of Annexin V and permeability to propidium iodide) showed no appreciable apoptosis or non-apoptotic cell death in either of U373 clones subjected to UV at 15 J/m^2 ^(not shown). The short term (up to 72 hours) cell proliferation was quantified using CCK-8 assay, and Figure 2C shows that proliferation after UV exposure was somewhat slower in U373cc3 cells, but the decrease was relatively minor. The colony formation after UV exposure was affected by CC3 only slightly, decreasing the number of colonies formed by U373cc3 cells by 5 to 10% compared to U373neo (not shown). Glioblastoma cells are notoriously resistant to death induced by DNA damage, and expression of CC3 apparently does not affect the apoptotic resistance of these cells.

### Silencing of CC3 has a modest effect on the repair of CPD lesions

**Figure 3:**
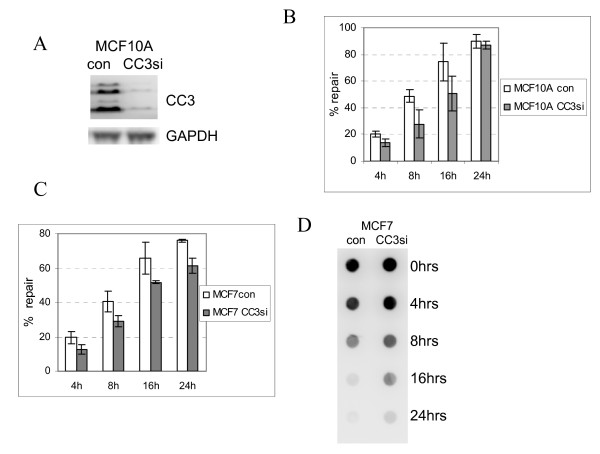
**Mild impairment of the repair of UV induced DNA lesions by abrogation of expression of endogenous CC3**. (A). Expression of CC3 in MCF10A cells infected with a control lentivirus and a CC3 siRNA expressing virus. (B). MCF10A cells were subjected to UVC irradiation at 10 J/m^2^, and harvested at different times after exposure. Relative repair of CPD lesions was quantified using ELISA with an anti-CPD antibody. Data represent average of three independent experiments. (C). A representative Southwestern blot analysis of DNA from UV-exposed MCF7 cells with anti-CPD antibody. (D) CPD repair in MCF7 cells, control and with silenced CC3. All charts show the average and standard deviation of three individual experiments.

We have next examined if silencing of CC3 expression affects the efficiency of DNA damage repair. Expression of CC3 was silenced in MCF10A cells, which derive from immortalized normal mammary epithelium (Additional File 1 contains information about all cell lines used in this study). These cells were infected with lentivirus expressing CC3 specific shRNA and puromycin resistance gene, and after selection the infected population showed very little residual expression of CC3 (Figure 3A). Examination of the fast branch of repair of the UV induced DNA damage (removal of 6-4PP) did not detect any significant differences in the rate of repair (not shown). However, repair of CPD as measured by ELISA showed a somewhat slower kinetic of CPD removal in MCF10A cells with silenced CC3, though at 24 hours similar percentages of CPD were repaired in both cell populations (Figure 3B). This was somewhat unexpected considering that the exogenously expressed CC3 has an inhibitory effect on repair of CPD lesions (Figure 2B).

To examine if the results obtained using MCF10A cells with silenced CC3 are unique to this cell line, we have silenced CC3 expression in a breast cancer cell line MCF7. We have again observed a modest delay in the removal of CPD (Figure 3C). To further confirm these results, we performed immunoblot analysis of DNA from UV-irradiated cells with a different anti-CPD antibody. As shown in Figure 3D, the delay in repair in cells with silenced CC3 was also detected by blotting assay in MCF7 cells, as well as in MCF10A cells (not shown). This confirms that the reduction in the levels of endogenous CC3 has a modest delaying effect on repair of UV induced DNA damage. This minor delay in the repair of CPD in absence of CC3 had no effect on the viability of cells after UV exposure (not shown). However, we have observed that silencing of CC3 had a negative effect on cell proliferation after UV exposure (see below).

### Changes in gene expression in cells forced to express CC3

To address the possible causes of the impaired repair of UV induced DNA damage we have conducted expression array analysis of U373neo and U373cc3 cells, before and after treatment with UV. The array analysis showed expression changes in a large number of transcripts in untreated U373cc3 cells compared to U373neo (Additional File 2). These transcripts represented genes that could be involved in a wide variety of cellular processes. Examination of genes induced by UV exposure showed that in general transcriptional responses to UV of U373 cells with or without CC3 expression are very similar; i.e. same mRNAs are induced or repressed in both cell lines, with a few exceptions (Additional File 3). This indicates that the transcriptional program induced by UV remains essentially unchanged in presence of exogenous CC3.

Two of the transcriptional changes observed in CC3 expressing cells versus parental U373 cells could be directly related to the differences in the UV induced DNA damage repair between the clones. Array results showed a rather high upregulation of the c-FOS transcript (13.2 fold) in untreated U373cc3 cells versus U373neo, and 2.7 fold downregulation of DDB2 (Additional File 2). c-FOS is an early response gene induced by UV [23], and was shown to be required for the efficient repair of the UV-induced DNA damage [24-26]. DDB2, also known as Xeroderma pigmentosum group E, is intimately involved in the nucleotide excision repair (NER) of DNA damage by recruiting ubiquitinating protein complex of DDB1-cullin 4A [27].

**Figure 4:**
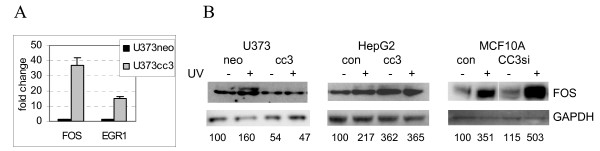
**CC3 negatively affects induction of c-FOS expression by UV**. A. Quantitative RT-PCR analysis of c-FOS and EGR1 expression in U373 clones. B. Western blot analysis of FOS expression in cells before and 2 hours after exposure to UV at 15 J/m^2^. The protein bands were quantified after scanning with the Kodak Imaging Station 2000R, and GAPDH levels were used to normalize the data.

Changes in the expression of the RNA for c-FOS were examined by quantitative RT-PCR. We have also examined expression of EGR1, an early response gene induced, among other treatments, by UV [28,29], because the array results showed upregulation of EGR-1 in untreated U373cc3 cells versus U373neo (Additional File 2). U373cc3 cells showed a major increase in transcription of c-FOS and EGR1 compared to U373neo (Figure 4A), but the protein levels of c-FOS (Figure 4B) and EGR-1 (not shown) in untreated U373 clones were not affected indicating that the increase in c-FOS transcription in U373cc3 cells does not results in the increase in protein levels. The array analysis also showed that UV induces c-FOS in U373neo cells (2. 5 fold) but not in U373cc3 (Supplemental File 2). Western blotting confirmed induction of c-FOS protein by UV only in U373neo cells (Figure 4B). To examine if CC3-induced inhibition of c-FOS induction by UV is unique to U373 cells, we have analyzed levels of c-FOS in CC3 null HepG2 cells transduced with a CC3 expressing lentivirus. Similar to U373 cells, exogenous CC3 also had a delaying effect on the repair of CPD lesions in transduced HepG2 cells (data not shown). Figure 4B shows that introduction of CC3 into HepG2 resulted in increase of basal c-FOS levels in untreated cells (not observed in U373cc3 cells even though RNA levels were higher), but, similar to the observations with U373 cells, prevented increase in c-FOS after UV exposure. These data indicate that exogenous CC3 might prevent induction of c-FOS by relatively low doses of UV (c-FOS was induced by UV of 40 and 60 J/m^2 ^in all cell lines; data not shown). We have not detected any effect of CC3 on subcellular localization of c-FOS which was exclusively nuclear in all cells (not shown).

We have then examined expression of c-FOS in cells with silenced CC3, expecting that perhaps the latter will have an enhancing effect on c-FOS induction by UV. Indeed, while there was no change in the basal levels of c-FOS in MCF10A cells with silenced CC3, induction of this protein by UV was much higher in MCF10A cells with silenced CC3 (Figure 4B). These observations suggest that both endogenous and exogenous CC3 might restrain induction of c-FOS by UV in different cellular contexts.

**Figure 5:**
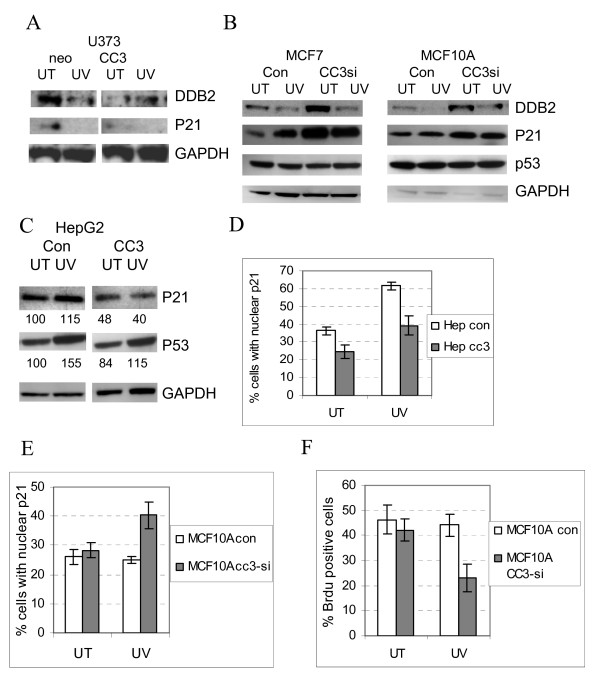
**Manipulation of CC3 expression affects expression levels of DDB2 and p21CIP1**. (A) Western blot analysis of U373 clones for expression levels of DDB2, p21CIP1 and GAPDH (loading control). Lysates were prepared from mock-treated cells and UV-treated (20 J/m^2^) cells at 2 hours after exposure. Cell lysates were electrophoresed and subjected to Western blotting with the antibodies to indicated proteins. (B). Western blot analysis of MCF10A and MCF7, control, and with silenced CC3, for expression levels of the indicated proteins. (C) Same as in (A) and (B) with HepG2 cells transduced with a control or CC3 expressing lentiviral vector. The protein bands were quantified after scanning with the Kodak Imaging Station 2000R with GAPDH signals used to normalize the data. (D) Subcellular localization of endogenous p21CIP1 in HepG2 cells with or without exogenously expressed CC3. Cells were stained for CC3 and p21CIP1 before and 2 hours after irradiation with UV (20 J/m^2^). The results are average of three experiments in which at least 300 cells were counted. (E). Localization of p21CIP1 in HepG2 cells before and 2 hours after irradiation with UV (20 J/m^2^). Experiments were done as in (D). (F) BrdU incorporation into MCF10A cells after exposure to 20 J/m^2 ^of UV. Cells were allowed to recover for 30 minutes after exposure, pulsed with 30 minutes with BrdU and incubated for further 1.5 hours in fresh media. After fixation, cells were stained with the anti-BrdU FITC conjugated antibody.

Next, we examined expression of DDB2 protein in cell lines where CC3 expression was manipulated. Figure 5A shows that the basal levels of DDB2 are lower in U373cc3 cells, in agreement with transcriptional downregulation found in the expression array analysis (Additional File 2). The basal levels of DDB2 protein were found to be higher in both MCF10A and MCF7 cells where CC3 was silenced (Figure 5B). These data indicate that CC3 has an inhibitory affect on expression of DDB2. We have examined the subcellular localization of DDB2 protein in various lines used above, but DDB2 was nuclear in all cells. We suggest that lower levels of DDB2 could be directly relevant to the delay in repair seen in U373cc3 cells.

### CC3 expression increases levels and nuclear localization of p21CIP1

In a recent publication, CC3 was reported to regulate levels of p53 and p21WAF1/CIP1 (CDKN1A) via stabilization of their mRNAs [30]. In addition, p21 protein levels have been shown to be regulated by DDB2 [31]. We have therefore examined if manipulation of CC3 affects the levels of p21CIP1 because p21CIP1 was shown to be involved in the UV induced DNA damage response in numerous publications. Western blot analysis of U373 cells for p21CIP1 showed somewhat lower levels in U373cc3 cells compared to U373neo, and disappearance of detectable p21 after UV exposure. We could not reliably examine the subcellular localization of p21CIP1 by immunofluorescence in U373 cells because these cells have very low levels of p21CIP1 most likely due to the lack of functional p53.

We have therefore examined levels and localization of p21 in CC3 protein null HepG2 cells that express p53. HepG2 cells were transduced with a CC3 expressing lentivirus (HepG2cc3) or a control vector (HepG2con). Expression of exogenous CC3 in HepG2 cells lead to a decrease in the basal levels of p21CIP1, and prevented the increase in its levels and nuclear localization after UV exposure observed in HepG2con cells (Figure 5C and 5D). Changes in levels and localization of p21CIP1 in cells with exogenously expressed CC3 are likely independent of p53-dependent regulation of p21, because the levels of p53 were only marginally affected by CC3 expression (Figure 5C), and the exclusively nuclear localization of p53 in HepG2 cells was not affected by CC3 (not shown).

Examination of MCF10A and MCF7 cells with silenced CC3 showed that in both cell lines abrogation of CC3 protein expression leads to increased basal levels of p21, but no further increase at 2 hours after UV exposure, unlike in control cells (Figure 5B). Silencing of CC3 did not affect the levels of p53 in cells before or 2 hours after UV exposure (Figure 5) (levels of p53 were increased at later times after UV exposure in both control and CC3-silenced cells; not shown). We have examined the subcellular localization of p21CIP1 in cells before and after UV irradiation. In untreated cells, similar percentages of cells had predominantly nuclear p21CIP1 irrespective of their CC3 status, but exposure to UV led to the nuclear accumulation of p21CIP1 in a higher percentage of CC3-silenced cells (Figure 5E). We have not detected changes in localization of p53 protein as a consequence of CC3 silencing (not shown). We conclude that lack of CC3 expression leads to increased nuclear localization of p21 after DNA damage.

Even though p21CIP1 has been implicated in regulation of DNA damage repair itself, some older and recent publications suggest that it is a negative regulator of the translesion (TSL) DNA synthesis after UV exposure. To examine if TSL is affected in MCF10cc3-si cells, we have examined the rate of DNA synthesis after UV exposure. Figure 5F shows that the number of cells in S phase after UV irradiation was significantly reduced in MCF10Acc3-si cells, which have higher levels of p21 that the control cells (Figure 4B). Considering that the effect of CC3 silencing on the efficiency of DNA repair was relatively minor in MCF10A and MCF7 cells (Figure 3), we conclude that the silencing of CC3 has a minor effect on the efficiency of DNA repair but strongly impairs the translesion DNA synthesis probably through increase in levels of nuclear p21.

### Exogenous CC3 affects repair of oxidative DNA damage

**Figure 6:**
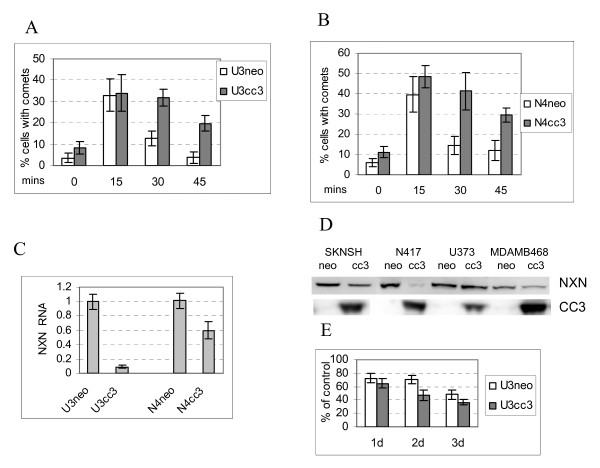
**CC3 impairs repair of oxidative DNA damage and reduces levels of nucleoredoxin RNA and protein**. (A). Comet assays were performed with U373neo and cc3 cells, either untreated (UT) or treated with 25 μM hydrogen peroxide for 15 mins, after witch peroxide was removed and cells were allowed to recover in fresh medium for additional 15 and 30 minutes. Results are average of three independent experiments. (B). Same as in (A), with N417neo and cc3 cells except the concentration of H_2_O_2 _was 10 μM. (C). Quantitative RT-PCR of steady-state NXN RNA levels was performed with RNAs isolated from the cells with or with exogenously introduced CC3. (D) Western blot analysis of nucleoredoxin expression in paired cells, control transfected (neo) or stably transfected with CC3. E. Viability of U373 clones subjected to treatment with 100 μM H_2_O_2 _for 30 minutes. Cells were analyzed as described in Materials and methods; experiments were performed three times with cells plated in triplicate or quadruplicate for each experimental point.

We have examined if repair of oxidative DNA damage is also affected by the exogenously introduced CC3 expression. U373 clones were treated with hydrogen peroxide for a short time and allowed to recover in fresh medium. Repair of oxidative damage was monitored by comet assay. Figure 6A shows that cells expressing CC3 have a decreased capacity for the repair of oxidative lesions in DNA. A similar deficiency in the repair of oxidative DNA damage was observed in a small cell lung carcinoma cells N417 (CC3-null) forced to express exogenous CC3 (Figure 6B).

The array expression analysis of U373 clones (Additional File 2) identified nucleoredoxin [32], a gene of the peroxiredoxin family, as downregulated 13.7 fold in U373cc3 cells. Nucleoredoxin (NXN) is involved in regulation of Wnt signaling pathway during oxidative stress and [33], and similar to the other members of the peroxiredoxin family, could be related to the control of the reactive oxygen species after oxidative insult. We examined levels of nucleoredoxin first by quantitative RT-PCR in U373 and N417 cell lines with and without CC3 and confirmed a very significant decrease in the transcript levels in presence of CC3 (Figure 6C).

Next, we have examined the levels of the nucleoredoxin protein by Western blot analysis. Unexpectedly, nucleoredoxin levels were not diminished in U373cc3 cells compared to U373neo cells in spite of the very significant reduction in RNA level (Figure 6D). Therefore, in U373 cells the impairment of the oxidative DNA damage repair is unlikely to be a direct or indirect consequence of the reduced levels of nucleoredoxin. In addition, the levels of both endogenous and peroxide treatment induced reactive oxygen species (ROS) in U373cc3 and U373neo cells were quantitatively similar, as measured by flow cytometric analysis of cells pre-loaded with ROS- sensitive probe CM-H_2_DCFDA (Molecular Probes; not shown). Analysis of cell survival after treatment with peroxide showed that it is diminished in U373 cells (Figure 6E), indicating that the delayed repair of oxidative damage might impact cell survival.

Because we have observed that in U373cc3 cells transcriptional changes are not necessarily "translated" into corresponding changes in protein levels (as seen with c-FOS, EGR1 and nucleoredoxin), we have examined expression of nucleoredoxin in other cells stably transfected with CC3. In N417cc3 cells nucleoredoxin protein was almost undetectable (Figure 6D). A significant reduction in nucleoredoxin protein levels was seen in two other lines forced to stably express exogenous CC3: CC3-negative neuroblastoma SKNSH and CC3-low breast carcinoma MDA MB 468 (Figure 5D). This suggests that suppression of nucleoredoxin expression by CC3 might be a common consequence of forced CC3 expression.

## Discussion

Our data show that the forced expression of exogenous CC3 and, to a much lesser degree, silencing of endogenous CC3 delay the repair of UV induced DNA damage. These findings are not entirely contradictory considering the role of CC3 in the regulation of nuclear transport. Changes in levels of CC3 could disrupt the existing balance in the nuclear import and export of any number of shuttling proteins, including those involved in DNA damage repair.

We have chosen to examine the possible consequences of forced expression of CC3 on DNA damage repair in a glioblastoma cell line lacking endogenous CC3, because introduction of CC3 did not affect its proliferation rate or sensitivity to death signals, unlike negative effects reported in many other tumor cell lines stably transfected with CC3 ([2]). Therefore, we were able to analyze effects of CC3 on DNA damage repair apart from the described negative effects of CC3 expression on cell survival and growth rate. In retrospect, however, we observed that some transcriptional changes induced by CC3 in U373 cells did not result in corresponding changes in protein levels (increases in c-FOS, EGR-1 and a decrease in nucleoredoxin). The lack of correlation between a change in a certain mRNA level and a corresponding change in level of protein it encodes appears to be limited to U373 cells. In particular, the FOS protein is induced by CC3 in HepG2 but not in U373 (Figure 4B); nucleoredoxin is downregulated by CC3 in three tumor cell lines but not in U373 (Figure 6D); vimentin, whose RNA is upregulated 300 fold in U373cc3 cells (Additional File 2) is practically unchanged at the protein level, but is strongly induced in N417 cells (data not shown). It appears that the U373 cells exercise a strong post-transcriptional control of protein levels in U373 cells, either at the level of translation per se, or by altering protein stability. This could be relevant to the ability of these cells to maintain levels of critical proteins that are compatible with the high proliferation rate and apoptotic resistance characteristic for glioblastoma.

We have attempted to identify some of the proteins relevant to DNA damage responses whose levels/localization are affected by changes in CC3 levels. The RNA expression array analysis was based on a rationale that CC3 might affect nucleocytoplasmic shuttling of certain transcription factors, which could have consequences for the expression levels of a number genes that are co-regulated by these transcription factors. In particular, we were interested to find if the introduction of CC3 into CC3-negative cells might have a generalized effect on transcription changes induced by UV. As seen in the Additional File 3, the sets of genes affected by UV in CC3-negative versus CC3-expressing cells are remarkably similar, excepting a few quantitative changes in the degree of suppression/induction. This indicates that CC3 expression does not induce a major shift in the transcriptional response to UV.

This is, perhaps, not surprising, considering that cells differing in CC3 expression could be expected to have significant changes in subcellular localization of the relevant shuttling proteins rather than changes in their mRNA levels. We have detected changes in the expression levels of two proteins closely related to NER: DDB2 and p21CIP1; in addition, the well-known induction of c-FOS by UV was significantly inhibited in CC3-expressing cells, and increased in CC3-silenced cells (Figure 4). The role of DDB2/XPE in NER has been studied extensively, but remains incompletely understood. Thus, degradation of DDB2 is thought to be essential for the initial stages of NER, but DDB complex activity is dispensable for the NER *in vitro *([34] and references therein). The prevailing notion was that DDB2 is an essential part of the UV-activated DDB1-DDB2-CUL4A ubiquitin ligase, and as such participates in the initiation of global NER. This complex was variously reported to be responsible for degradation of DDB2 itself [35] or ubiquitination of histones at the sites of damage [36,37]. However, a very recent report showed that ablation of Cul4A, a ubiquitin ligase in the complex, actually increases the capacity of normal cells to repair UV damaged DNA [38]. DDB2 was shown to act as a haploinsufficient tumor suppressor [39], but to complicate the issue further, DDB2 is expressed at higher levels in estrogen receptor positive breast cancer cells, and its knockdown negatively impacts the growth rate of these cells [40]. This, however, could be due to the functions that DDB2 likely has in cellular processes other than NER. We show that DDB2 levels are increased in MCF10A and MCF7 cells when CC3 expression is silenced, and DDB2 levels are decreased in U373 cells forced to express CC3. The latter change correlates with a slower kinetics of UV induced DNA damage repair. We conclude that expression of CC3 has an inhibitory effect on expression of DDB2, and that could play a role in the delay of DNA repair.

The role of p21CIP1WAF/CIP1 in NER remains controversial, in spite of hundreds of research papers published on the topic. Earlier reports indicated that higher levels of p21CIP1 are not inhibitory for NER, but can inhibit translesion DNA synthesis (TLS) [41], while subsequent publications suggested an inhibitory role of p21 in the repair process itself via its interaction with PCNA (reviewed in [42]). p21 was strongly implicated in the negative regulation of TLS, preventing association of polymerase eta with PCNA [43-45]. Our findings show that increased levels of p21CIP1 in cells where CC3 is silenced correlate with the significant inhibition of TLS, but have a minor effect on UV induced DNA damage repair.

**Figure 7:**
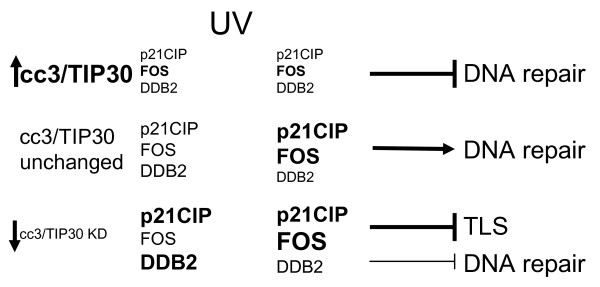
**Schematic illustration of the proposed/expected mechanism of the effects of CC3/TIP30 on responses to DNA damage**. The scheme illustrates changes in expression of thee proteins involved in DNA damage responses depending on changes in levels of CC3. Changes in protein levels are represented as increases/decreases in the font size or thickness compared to the uniform size of font used for all proteins in the middle part of the scheme ("CC3/TIP30 unchanged") prior to UV treatment. Overexpression of CC3 (top part) leads to higher levels of c-FOS and lower levels of p21 and DDB2, while silencing of CC3 has no effect on c-FOS in untreated cells, but significantly increases levels of DDB2 and p21CIP. After exposure to UV cells expressing exogenous CC3 protein fail to increase levels of FOS and p21CIP. They also accumulate less p21CIP in their nuclei after UV exposure (Figure 5D). This could contribute to the deficiency in repair of DNA damage. Cells where CC3 expression is silenced (lower part. "CC3/TIP30 KD) continue to maintain the already higher levels of p21CIP without further increasing it. More of these cells have nuclear p21 (Figure 5E). They also show a higher increase in the levels of FOS after UV exposure. Altogether, these changes observed after CC3 knockdown have minor consequences for the repair of DNA damage, but significantly inhibit DNA translesion synthesis (TLS) after DNA damage likely due to high levels of p21CIP.

Expression of c-FOS was shown to be necessary for the efficient repair of UV-induced DNA damage [24,25], and c-FOS negative cells show a significant decrease in their repair capacity. Two cell lines expressing exogenously introduced CC3 (U373 and HepG2) fail to increase expression of c-FOS after UV exposure, which could be a contributing factor to the observed delay in repair. Our observations of the complex effects that manipulation of CC3 levels has on levels of the three investigated proteins relevant to DNA damage responses are illustrated in Figure 7.

We have also investigated the effect of manipulating levels of CC3 on the DNA damage repair and cell survival after oxidative stress. While we could not detect any effects of CC3 knockdown on the repair of oxidative DNA damage, excess of cellular CC3 significantly delayed repair, and, in addition, had a negative effect on cell proliferation. These findings are in agreement with the published results that overexpression of CC3 leads to increased sensitivity to the apoptotic induction by oxidative stress [30]. An increase in levels of p53 and p21 proteins trough the stabilization of the p53 RNA was suggested to play a role in predisposition to apoptosis induced by CC3 expression.

It is possible that the delays in repair of oxidative DNA damage induced by CC3 might contribute to the lower survival of glioblastoma cells expressing exogenous CC3 (Figure 6E). Other cell lines forced to express CC3 (Figure 6D) also show an impaired survival after treatment with oxidative agents (not shown). In these cell lines, the negative effect on viability could be due not only to impaired DNA repair, but also to a decrease in levels of nucleoredoxin, induced by CC3 (Figure 5). Lower levels of nucleoredoxin in cells forced to express CC3 could result in lowered ability to neutralize ROS, more severe DNA and other types of oxidative damage, and consequently more death. Indeed, N417 cells expressing exogenous CC3 have higher levels of endogenous ROS as measured by conversion of ROS-sensitive probe CM-H_2_DCFDA to fluorescent form (data not shown). The possible role of nucleoredoxin in this process has not been studied, but peroxiredoxins in general have been implicated in the ability of both Saccharomyces cerevisiae [46] and mammalian cells [47] to protect themselves from the deleterious effects of oxidative insults on DNA repair, genomic stability and survival. Nucleoredoxin was shown to act as a negative regulator of the Wnt/β-catenin signaling in peroxide-treated cells [33]. The long-term consequences of this effect on cell survival have not been investigated. However, assuming that nucleoredoxin shares with other peroxiredoxins the ability to control ROS levels, it is reasonable to suggest that lowering levels of cellular nucleoredoxin will have an adverse effect on cell survival after oxidative insult.

## Conclusions

Our experiments show that exogenously expressed CC3, by virtue of its effects on levels and subcellular localization of proteins involved in DNA damage responses, negatively affects DNA damage repair after both UV exposure and oxidative stress. We have identified c-FOS, DDB2 and p21CIP as proteins whose levels or localization are affected by CC3, and these changes are potential culprits in the effects of CC3 on cellular responses to UV. Our findings are consistent with the known role of CC3/TIP30 in impairing cell survival after apoptotic treatments and its role as a tumor and metastasis suppressor. Inhibition of nuclear transport by CC3 most likely plays a role in its activity as metastasis suppressor and cell death promoter. Further work will be needed to identify critical proteins whose localization is affected by CC3 and impacts responses to DNA damage.

## Methods

### Cells, transduction and transfection

All cell lines were obtained from the ATCC, and propagated according to the instructions provided. SiRNA constructs in the LKO plasmid vector for the production of lentivirus - mediated siRNA expression were purchased from Open Biosystems/Thermo. Viruses were produced in HEK293 cells according to the manufacturer's instructions and used to transduce cells, followed by selection in pre-determined concentration of puromycin. Stably transfected clonal populations of U373, N417 and SKNSH cells were selected and described earlier [2,3].

### HCR assay

The HCR assay was conducted as described previously [48]. The firefly luciferase reporter plasmid (pFLuc) either damaged with 1000 J/m^2 ^or undamaged was contransfected with undamaged Renilla luciferase pRLuc as an internal transfection control and with effector plasmids: pCMVneo3, pCMV-CC3 and pCMV-CC3 mut with Fugene (Roche) on 24 well plates. At 24 hours after transfection, cells were lyzed and assayed for Renilla and Firefly luciferase activity using the Dual Luciferase Activity assay (Promega). The firefly luciferase activity of each sample was normalized to the Renilla activity. To determine the repair capacity of cells transfected with different reporters, repair conversion was calculated first by dividing the normalized firefly luciferase activity from cells transfected with the UV-damaged pFLuc by that of undamaged pFLuc transfected cultures. The fold HCR was calculated by dividing the repair conversion of effector transfectants by that of vector transfectants.

### Comet assay

Comet assays were conducted using Trevigen comet assay kits according to manufacturer's instructions. Briefly, cells were harvested by trypsinization at different times after treatment with UV or hydrogen peroxide, and embedded into low-melting agarose. Embedded cells were gently lyzed, and the nuclear DNA was denatured with alkali. Cells in agarose were subjected to electrophoresis in the alkaline buffer, and after drying were stained with SYBRgreen. Comets were counted under fluorescent microscope. At least 300 nuclei were counted for each sample.

### Detection of CPDs by ELISA

Cells were seeded on 6-cm culture dishes at 5 × 105 per well and irradiated next day with 15 J/m2

UVC. Cells were harvested immediately or at different times after irradiation, and genomic DNA was isolated (DNeasy cell and tissue kit; Qiagen). ELISA (in triplicate) was performed in 96-well plates precoated with 0.06% protamine sulfate (Sigma). For CPD detection with TDM-2 antibody (Cosmo Bio), 30 ng of heat-denatured DNA in PBS was added to the wells and dried at 37°C for 16-20 h. The monoclonal antibody TDM-2 was added to the wells at 0.05 μg/ml and incubated at 37°C for 30 min. The plates then were incubated with affinity-purified goat anti-mouse immunogloblin G conjugated with peroxidase (Zymed) at 37°C for 90 min. Finally, the substrate solution, consisting of 0.04% o-phenylene diamine and 0.007% H_2_O_2 _in citrate-phosphate buffer, was added to each well for 30 minutes. Sulfuric acid was added to stop the reaction and the absorbance at 490 nm was measured using a Microplate Reader Spectra MAX.

### Detection of CPDs by Immuno-slot blot

100 ng of DNA was diluted into DNA denaturation solution (1.5 M NaCl: 0.5 M NaOH) and applied to a positively charged nylon membrane by vacuum blotting. The DNA on membrane was washed with a neutralizing buffer (0.5 M Tris; 1.0 M NaCl, pH 7.5). The membrane was baked at 80°C for 20 minutes and blocked by incubating in PBS plus 0.1% Tween 20 (PBS-T) containing 5% nonfat milk (blocking buffer) overnight at 4°C. After multiple washes with PBS-T, the membrane was incubated with anti-CPD antibody (Kamiya) in blocking buffer for 2 h at room temperature. The membrane was washed thoroughly with PBS-T and further incubated with an anti-mouse horseradish peroxidase-conjugated antibody (Pierce) for 1 h at room temperature (1:5,000 dilution in PBS-T). The membrane was developed with the chemiluminescence detection kit (Pierce) and detected on Kodak Imager 2000R using Kodak Molecular Imaging software.

### Cell viability tests

Short term effects of various treatments were analyzed using the WST-8 cell proliferation assay with the Cell counting kit CCK-8 from Dojindo. Briefly, cells were plated on 96 well palates, subjected to treatments in triplicates or quadruplicates, and the number of live cells was evaluated one, two and three days later. For colony-forming assay, cells were plated at 1000 on 6 cm plates overnight, and subjected to UV exposure. Fifteen days later the colonies were stained with Crystal Violet and counted.

### Western blotting

Whole cell lysates were prepared in RIPA buffer (140 mM NaCl, 50 mM Tris pH 8.0, 1 mM EDTA. 1 mM DTT, 0,5% deoxycholate, 0.1% SDS, 1% Triton X100 and protease inhibitors). For analysis of DDB2, RIPA buffer contained 420 mM NaCl. Cell lysates were electrophoresed on SDS-PAGE and transferred to nitrocellulose membranes. Membranes were blotted with antibodies at recommended concentrations overnight at 4°C and the bound primary antibodies were detected using peroxidase-conjugated secondary antibodies. Blots were developed using SuperSignal enhanced chemiluminescence kit (Pierce) and imaged on Kodak Imager ISR2000.

### RNA isolation and microarray analysis

U373neo and U373cc3 were exposed to UV at 15 J/m^2 ^and left to recover for 2 hours. These cells, along with untreated cells, were each harvested in triplicates for microarray experiment. Total RNA was isolated using RNeasy Mini Kit (Qiagen). RNA was used for Cy5-labeled aRNA preparation per manufacturer instruction (Ambion MessageAmp™ aRNA Amplification Kit, Ambion Inc., Austin, TX, USA).

The microarray experiments were performed using Phalanx Human OneArray™ Version 4.1 (HOA 4.1; Phalanx Biotech Group, Inc., Hsinchu, Taiwan). Each microarray contains 32,050 oligonucleotide probes that include 30,968 human gene probes for transcription expression profiling and 1082 experimental control probes. Detailed descriptions of the gene array list, hybridization and processing procedures are available from http://www.phalanxbiotech.com/.

In brief, each Cy5 labeled aRNA was hybridized to HOA 4.1 microarrays in triplicate. Prior to the microarray hybridization, the Cy5 labeled aRNAs were fragmented using the reagents and protocol provided in Ambion RNA Fragmentation Reagents kit (Ambion Inc., Austin, TX). Fragmented Cy5-labeled aRNA were suspended in OneArray™ hybridization buffer (provided in HOA product package) at a final volume of 180 μl per hybridization. The pre-hybridization blocking, array hybridization, and post-hybridization washes were performed according to the instruction provided in the HOA User Guide. The arrays were then scanned using GenePix 4000B (Molecular Devices, Sunnyvale, CA, USA) and the fluorescent intensities were extracted from the generated images by following the instructions and the conditions described in HOA User Guide. The raw intensity data were input to GeneSpring GX Version 7.3.1 (Agilent, Foster City, CA, USA). The averaged intensities for each probe then were normalized by quantile normalization. Differentially expressed genes were identified, and filtered based on t-tests (p < 0.05). The results of microarray analysis are available at http://www.ncbi.nlm.nih.gov/geo/query/acc.cgi?acc=GSE20633

### Real-Time Quantitative PCR

Total RNA was isolated as described above. Five μg of total RNA was treated with 20 units of DNase and reverse transcribed with oligo dT:random primer (1:1 at 1.5 μg each) by using Superscript II (Invitrogen). Fifty nanograms of first-strand cDNA were used in subsequent real-time PCR carried out with iQ5 (Bio-Rad) by using SyBr green dye (Applied Biosystems) as the fluorescent probe with gene-specific primer sets. The following parameters were used in the PCR: 10 min denaturation at 95°C, 35 cycles of 15 sec at 95°C, and 1 min at 55°C. To quantify fold expression changes compared to control, the ΔΔC^T ^methods were used [49]. C^T ^is the point at which the fluorescence signal rises above the baseline fluorescence and begins to increase exponentially. The C^T ^value is in logarithmic inverse relationship with the abundance of the transcripts, based on the assumption that C^T ^values increase by 1 for each twofold dilution.

### Immunofluorescent staining

Cells were plated on 8-well chamber slides, treated as intended next day and fixed in 4% paraformaldehyde. After permeabilization with 0.1% Triton X-100 and blocking in 3% BSA in PBS, cells were incubated with anti-p21CIP1 antibodies (Cell Signaling) and anti-CC3 antibodies [18] in 1% BSA, and bound antibodies detected with the appropriate secondary antibodies conjugated to Alexa 488 or 568 (Invitrogen).

### BrdU incorporation

Cells in culture were incubated with 30 μM of BrdU for 30 minutes, after which BrdU containing media were removed, cells washed with PBS and incubated for additional 1.5 hours in fresh media. Cells were harvested, fixed in 70% ethanol and processed for staining with anti-BrdU, FITC-conjugated antibody (Becton Dickinson) according to supplier's protocol. Cells were counterstained with propidium iodide and analyzed on FACScan.

## Competing interests

The authors declare that they have no competing interests.

## Authors' contributions

SF and FK carried out manipulations of cell lines' CC3 content and analysis of proteins and RNA expression. SF performed the expression array analysis. ES performed DNA damage repair assays and cell staining, designed the study and wrote the manuscript. All authors read and approved the final manuscript.

## Supplementary Material

Additional file 1 **Summary of changes induced in cell lines by manipulation of CC3 expression**. The Table shows list of cell lines used in the study including the status of endogenous CC3 expression, the effects of manipulating CC3 levels on cell proliferation and survival, and changes in expression levels of DDB2, p21CIP, FOS and NXN in response to changes in CC3 levels. Click here for file

Additional file 2 **List of genes differentially expressed in untreated U373neo cells versus U373cc3 cells**. The Table shows results of comparative expression array analysis of the glioblastoma cells U373 stably transfected with CC3 (U373cc9) versus control transfected U373 neo. Transcripts upregulated in U373cc9 cells are shown in the first (upper) part of the Table, and transcripts downregulated in U373cc3 versus U373neo are shown in the second part of the Table. Click here for file

Additional file 3 **List of genes induced or repressed after UV exposure in U373 clones with or without exogenous CC3**. The Table shows expression changes induce by UV in glioblastoma clones. U373neo and U373cc3 cells were exposed to 20 J/m^2 ^UV. RNAs were prepared two hours later. Transcripts induced and repressed by UV are listed; ranking was based on U373neo cells. Click here for file
