# Supplementary material for: CC3/TIP30 affects DNA damage repair
Source: BMC Cell Biol. 2010 Apr 7;11:23. doi: 10.1186/1471-2121-11-23 (PMC2867790; doi:10.1186/1471-2121-11-23)
Supplement: Additional file 1 — Summary of changes induced in cell lines by manipulation of CC3 expression. The Table shows list of cell lines used in the study including the status of endogenous CC3 expression, the effects of manipulating CC3 levels on cell proliferation and survival, and changes in expression levels of DDB2, p21CIP, FOS and NXN in response to changes in CC3 levels. [file 1471-2121-11-23-S1.DOC]

|  | U373 | HepG2 | MCF7 | MCF10A | N417 | SKNSH | MDAMB468 |
| --- | --- | --- | --- | --- | --- | --- | --- |
| Cell line origin | glioblastoma | hepatoma | breast carcinoma | immortalized  mammary epithelium hepatoma | small cell lung carcinoma | neuroblastoma | breast  carcinoma |
| Endogenous CC3 | none | none | expressed | expressed | none | none | very low |
| Change in CC3 levels performed | overexpression | silencing | silencing | overexpression | overexpression | overexpression | overexpression |
| Effect of CC3 overexpression or silencing on proliferation or survival | none | none | none | none | Inhibition of cell proliferation and predisposition to apoptosis | Inhibition of cell proliferation and predisposition to apoptosis | Inhibition of cell proliferation and predisposition to apoptosis |
| DDB2 expression | decrease | ND | increase | increase |  |  |  |
| P21CIP expression | decrease | decrease | increase | increase |  |  |  |
| FOS expression | No change in untreated cells;  no induction by UV | Increase in untreated; no induction by UV | ND | No change in untreated; higher induction by UV |  |  |  |
| NXN | No change | ND | No change | ND | decrease | decrease | decrease |
